# The role of supportive supervision using mobile technology in monitoring and guiding program performance: a case study in Nigeria, 2015–2016

**DOI:** 10.1186/s12889-018-6189-8

**Published:** 2018-12-13

**Authors:** Sisay G. Tegegne, Faisal Shuaib, Fiona Braka, Pascal Mkanda, Tesfaye B. Erbeto, Aron Aregay, Oyaole D. Rasheed, Akpan G. Ubong, Njie Alpha, Ahmed Khedr, Mirghani A. Isameldin, Yared G. Yehushualet, Charity Warigon, Usman Adamu, Eunice Damisa, Bassey Okposen, Peter Nsubuga, Rui G. Vaz, Wondimagegnehu Alemu

**Affiliations:** 1 World Health Organization Country Office, Abuja, Nigeria; 2 National Primary Health Care Development Agency, Abuja, Nigeria; 3 World Health Organization, Regional Office for Africa, Brazzaville, Congo; 4 Global Public Health Care Solutions, Atlanta, GA USA; 5 World Health Organization, Headquarters, Geneva, Switzerland

**Keywords:** Real-time data, Supportive supervision, Timely feedback, Health facility distance

## Abstract

**Background:**

Supportive supervision is one of the interventions that fosters program improvement by way of imparting knowledge and skills to health workers. The basic challenge in supportive supervision is the availability of data in real time for timely and effective feedback. Thus, the main objective of this study was to determine the contribution of real-time data collection during supportive supervision for timely feedback and generation of evidence for health intervention planning.

**Methods:**

We analyzed supportive supervision records collected through handheld devices employing the open data kit (ODK) platform from July 2015 to June 2016. Supervision was conducted across the country by 592 World Health Organization (WHO) officers. The availability of real-time data and the distance of health facilities to the community were analyzed.

**Results:**

During the study period, 90,396 health facilities were supervised. The average time spent during supervision varied from 1.53 to 3.78 h across the six geopolitical zones of the country. The average interval between completion of the supervisory checklist and synchronization with the server varied from 3.9 h to 7.5 h. The average distance between the health facility and a ward varied from 5 to 24 km.

**Conclusion:**

The use of handheld devices for supportive supervision provided real-time data from health facilities to state and zonal levels for analysis and feedback. Program officers used the findings to rectify process indicators in time for a better outcome.

## Background

Supportive supervision is an effective strategy for continuously enhancing staff performance. It is carried out with the focus on using supervisory visits as an opportunity to improve the knowledge and skills of health staff [1]. A study on the rise and fall of supervision in a project designed to strengthen supervision of the integrated management of childhood illness in Benin emphasized the importance of supervision for management purposes. The study suggested that resources should be allocated to promote supervision and to remove the obstacles of supervision [2].

The importance of supportive supervision in public health has been linked with improvements in program performance. A study in Zimbabwe showed that, following supervision, overall drug management improved significantly compared with control groups. Similarly, a study in India showed that regular supervision of sessions and vaccine stores are an important step in ensuring quality immunization services [3, 4].

Another study conducted in Nigeria, on supportive supervision as an effective intervention in achieving high-quality malaria case management at the primary healthcare level, recommended that supportive supervision should be incorporated into existing frameworks for improving healthcare worker performance [5].

Furthermore, a desk review of publications and gray literature on community health worker effectiveness showed that supportive supervision strengthened the productivity of community health workers [6].

However, there is little evidence on technology-assisted mobile data collection for real-time data transfer during supportive supervision. However, evidence has been found for the effect of the modality of supervision on its quality. For instance, a study on the use of personal digital assistants for data entry in southern Tanzania showed that electronic methods of data collection were efficient for data entry and merging [7].

Similarly, mobile technology in health interventions has shown a change in program management in southern Zambia. In another study, mobile phones were used for reactive case searches through timely and accurate dissemination of local surveillance information [8].

The importance of timely data availability for prompt case detection and subsequent case management was found to be beneficial in a study in Pakistan. The study showed that the information exchange using mobile phones improved supportive supervision by enhancing coordination among health workers for timely case reporting and appropriate follow-up [9].

The experience with a large-scale baseline survey on research conducted on the use of mobile phones as a data collection tool in South Africa suggests that the system is preferable to a paper-based approach. The real-time quality control and supervision of data collection that are enabled by the use of a mobile phone-based survey system makes this an attractive management option [10].

The added advantage of using mobile phones for collecting data during health facility supervision is the availability of the global positioning system (GPS) within current generation mobile phones or smartphones. Using GPS information, it is possible to obtain approximate distances between healthcare facilities, which could inform proper health service provision. A study performed in Kenya on modeling distances traveled to government health services showed a link between the achievement of Millennium Development Goals and access to health facilities [11]. Various studies have also shown that knowledge of the actual distance of health facilities from each other has policy implications. A study in Nigeria showed that the distance to primary healthcare facilities was a barrier to proper utilization of health facilities. An assessment of the factors associated with utilization of immunization services in the community in a rural setting in Ethiopia showed that among the factors significantly associated with full immunization of children was the average walking time from home to health facilities. In families where the average distance was < 1 h the immunization coverage was higher [12–14].

We set out to determine the contribution of supportive supervision using mobile technology in providing timely program information in the polio program in Nigeria. We also measured the distance of the health facility to be accessed by the communities from the information gathered. We also identified how supportive supervision contributes to the implementation of other non-polio health programs in visited facilities.

## Methods

### Outline of study method

We analyzed supportive supervision findings collected by mobile phones under open data kit (ODK) platform from July 2015 to June 2016. ODK is a free and open-source set of tools. ODK provides an out-of-the-box solution for users to build a data collection form or survey, collect the data on a mobile device, send it to a server, aggregate the collected data on a server, and extract it in useful formats [15].

The checklist for supportive supervision was uploaded to the mobile phones of World Health Organization (WHO) officers. The officers used this checklist to supervise health facilities. The system replaced a paper-based data collection method. After data had been collected at the field level, it was transferred to the central server. The data was then downloaded from the server at the national and zonal level in real time. The ODK platform has inbuilt data analysis for prompt feedback. We measured the time lapse from data capture to server upload to measure the availability of data for action in real time. A time stamp was also embedded in the platform to measure the time taken to perform supportive supervision. We analyzed the maximum, minimum, and average time spent measuring the quality of supportive supervision.

A GPS coordinate was captured at the beginning and end of supportive supervision to measure the actual geolocation of the facilities visited. Using the GPS coordinates, we mapped the distance of each health facility from the Local Government Area (LGA) headquarters and the nearest ward for the health facility supervised. We also performed trend analysis of the collected information on surveillance and routine immunization over the study period.

Supervision was conducted across the country by 592 WHO officers. All officers were provided with mobile phones and given access to download a form for use during supervision. At the end of each supervisory visit, the officers sent the data to a centrally managed server.

Although supervision took place in all health facilities based on surveillance prioritization, we analyzed supervisory data collected from all health facilities that conducted routine immunization in Nigeria during July 2015 to June 2016. We also measured the contribution of polio structure in visited facilities for strengthening routine immunization.

### Procedures

A structured questionnaire was developed that consisted of general information on visited facilities, disease surveillance, and routine immunization activities. The questionnaire was pretested in Kano state to verify the accuracy and flow of the questions and to assess the average time required to supervise a health facility. Time measurements and GPS coordinates were included in the checklist. We provided hands-on training for 592 WHO field officers on how to download a questionnaire on their mobile phones from a server and on how to send a completed questionnaire back to a server. Data managers in the six geopolitical zones of the country (namely north-central, northeast, northwest, southeast, southwest, and south-south zones) were trained to assist in the process. We also provided training for troubleshooting during the process of data collection.

### Data analysis

All data sent to the server were downloaded at the zonal and national levels for detailed analysis and feedback. Regular feedback was provided to all officers. Furthermore, the ODK platform was customized to produce instant analysis of each question in the supervisory checklist for onsite feedback. The time taken to administer the entire checklist was also analyzed.

## Results

A total of 90,396 facilities were supervised at least once during the study period from July 2015 to June 2016. Of the total supervisions conducted based on priority levels, 86% were providing routine immunization services. During the supportive supervision, geocoordinates of health facilities were captured. The precision of actual health facility location captured with the system was within a radius of 100 m.

The average time to conduct supervision varied from 1.53 to 3.78 h across the six geopolitical zones of the country. The national average time was 1 h 99 min. The average time to send the completed checklist of supportive supervision to a central server once supervision was completed varied from 3.9 to 7.5 h. The national average time for submitting the data to the server as a measure of real time submission was 5.28 h (Table 1).

**Table 1:** Average time of supportive supervision verses time to submit to server, July 2015 to June 2016, Nigeria

Zones	Average time of supervision in the field (h)	Average time submitted to server (h)
North-central zone	2.42	4.5
Northeast zone	2.05	7.5
Northwest zone	1.53	7.3
Southeast zone	2.76	4.3
South-south zone	3.78	4.2
Southwest zone	2.57	3.9
National	1.99	5.28

Much of the supportive supervision by WHO officers was jointly conducted with government counterparts. As shown in Table 2, the proportion of joint supervision with government counterparts was above 50% for each month during the study period. The highest proportion of joint supervision was 57%.

**Table 2:** Proportion of supportive supervision conducted with government counterparts, July 2015 to June 2016, Nigeria

Month	Proportion of joint supervisions
July 2015	55%
August 2015	54%
September 2015	53%
October 2015	51%
November 2015	51%
December 2015	48%
January 2016	56%
February 2016	54%
March 2016	56%
April 2016	54%
May 2016	54%
June 2016	57%

**Fig. 1:**
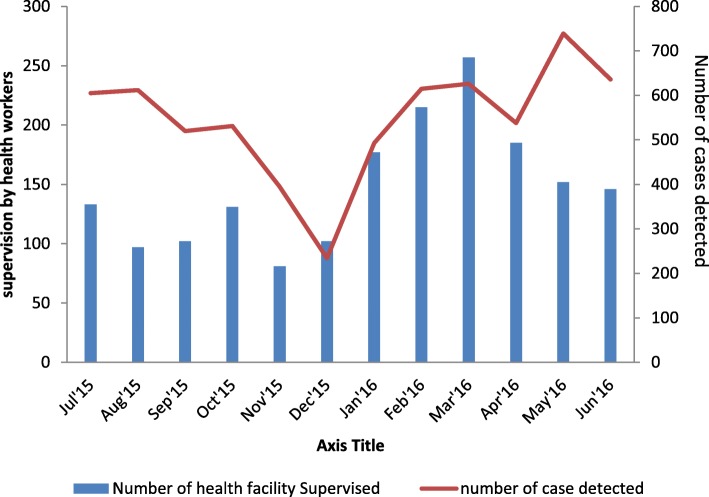
Number of supportive supervision to health facilities with the number of AFP cases detected by health workers, July 2015 to June 2016, Nigeria

As shown in Fig. 1, acute flaccid paralysis (AFP) cases were detected in health facilities that benefited from supportive supervision during the study period. The analysis excluded health facilities that had conducted training during the study period. The only opportunity for the health workers in those facilities was sensitization during supportive supervision. Of the total 6544 AFP cases detected during the study period, 1778 were in health facilities by focal persons that were not formally trained but were supervised. The rest of the cases were from focal persons that were trained and supervised during the year.

The average distance of health facility to a ward varied from 5 to 24 km. The maximum was in the northeast geopolitical zone, while the lowest average distance of health facilities to a ward was in the southwest geopolitical zone. The national average distance between wards and health facilities was 13 km (Table 3).

**Table 3:** Average distance of health facilities to nearest ward in kilometers by state; analysis of supervisory data using mobile phones, July 2015 to June 2016, Nigeria

Zone	State	Average distance accessing facilities (km)
North-central zone	Benue	17
	FCT, Abuja	15
	Kogi	12
	Kwara	17
	Nasarawa	24
	Niger	22
	Plateau	16
Zone average		18
Northeast zone	Adamawa	21
	Bauchi	21
	Borno	14
	Gombe	13
	Taraba	22
	Yobe	24
Zone average		20
Northwest zone	Jigawa	16
	Kaduna	20
	Kano	11
	Katsina	13
	Kebbi	16
	Sokoto	17
	Zamfara	19
Zone average		16
Southeast zone	Abia	8
	Anambra	7
	Ebonyi	11
	Enugu	12
	Imo	6
Zone average		9
South-south zone	Akwa Ibom	8
	Bayelsa	13
	Cross River	17
	Delta	11
	Edo	17
	Rivers	9
Zone average		12
Southwest zone	Ekiti	5
	Lagos	6
	Ogun	10
	Ondo	11
	Osun	5
	Oyo	8
Zone average		7
National		13

**Fig. 2:**
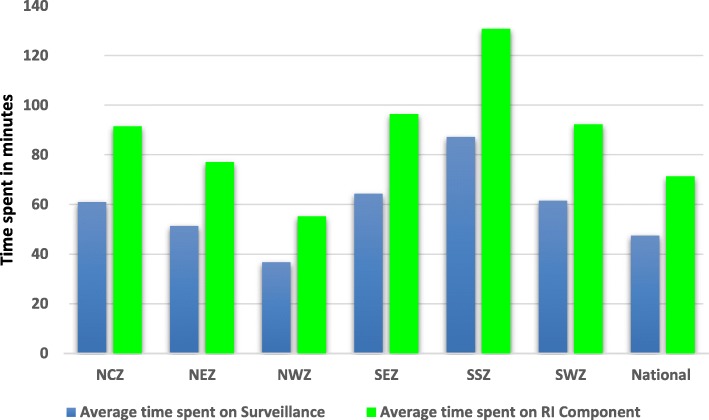
Average time spent on supportive supervision for routine immunization (RI) and surveillance; supportive supervision data 2015–2016, Nigeria. NCZ north-central zone, NEZ northeast zone, NWZ northwest zone, SEZ southeast zone, SSZ south-south zone, SWZ southwest zone

The average time spent at the national level was 71 min for the routine immunization section of the supportive supervision, while for surveillance the average was 48 min. Among the six geopolitical zones in the country, the lowest average time spent for supportive supervision was 55 min for routine immunization and 37 min for surveillance. The northwest had the lowest record of time spent for supervisory support, while the south-south zone had the highest record. (Fig. 2).

## Discussion

We sought to determine the real-time data availability for timely feedback using mobile phone-assisted supportive supervision. We found that supportive supervision, using a handheld mobile data collection tool, was performed on average in 199 min and transferred to a central level server within 5 h from more than 6000 health facilities across the country. The evidence suggests that real-time data were available for action at the national and zonal levels on the same day that supervision took place in health facilities. Program officers used the findings to rectify process indicators in time for a better outcome.

We found out that health workers detected AFP cases, and the detection correlated with the real-time supportive supervision to the reporting health facilities. We disaggregated the data to look at the pattern of reported AFP cases in facilities that have not received formal training but received supervision as the only means of sensitization to detect cases. The detection of cases was likely due to sensitization during the supportive supervision of focal persons.

Ours is not the only study to demonstrate a correlation between supportive supervision and change in outcome. A case study on supportive supervision on monitoring and evaluation in Haiti by Marshall and Fehringer [16] showed that the contribution of supportive supervision had a similar result on health worker productivity. A similar study by Frimpong et al. in Ghana [17] demonstrated that investment in supportive supervision could help maximize the output of scarce human resources in primary healthcare facilities.

We also observed that in all six geopolitical zones of the country more time was spent in supervising routine immunization activity than on surveillance. As a result of this time spent in supervising routine immunization activities, improvements in process indicators such as a steady increase in the availability of updated monitoring charts for routine immunization, recording and reporting tools, and availability of supplies for routine immunization were observed. This finding is in line with Reaching Every District (RED) approach where supportive supervision is a component for improving routine immunization [18]. Also, this finding could be used for mainstreaming the polio functions to routine immunization activities.

The findings of distances of health facilities to be accessed by the community could be used to plan health interventions to the community. A similar finding was recorded in a study conducted in Bangladesh on a cost-minimization approach to planning the geographical distribution of health facilities [19]. The study identified the distance to a health center as the most important factor determining utilization.

One of the limitations of our study was the association between improvement in surveillance and routine immunization indicators with supportive supervision. Improved program output could also be associated with training of health workers and sensitization sessions. Although it is difficult to control all contributing factors, we did exclude all health facilities in the analysis where training for health workers took place 1 year prior to the study period.

## Conclusions

Supervision using mobile technology has contributed to producing real-time data. The system has been used to locate the actual location of facilities and the distance of a health facility from a given point. It helps to give timely feedback and plan health interventions properly. The system of using mobile phones could be utilized to monitor program implementation for other health interventions such as defaulter tracing in routine immunization, sending alerts for outbreak-prone diseases, investigation of outbreaks, and supervision of any other health interventions.

We recommend that a study be conducted to assess the impact of utilization of inbuilt feedback by supervisors to give real-time feedback to each health facility supervised at the point of supervision.

## Abbreviations

AFP: Acute flaccid paralysis
GPS: Global positioning system
LGA: Local Government Area
ODK: Open data kit
RED: Reaching every district
WHO: World Health Organization
